# How expressive ties energize competitive performance in DanceSport dyads: unraveling the role of athlete engagement in an innovatively applied actor-partner interdependence mediation model

**DOI:** 10.3389/fpsyg.2024.1412596

**Published:** 2024-05-27

**Authors:** Xiuxia Liu, Yang Liu, Dandan Pan, Xinghe Weng

**Affiliations:** ^1^Department of Physical Education, Xiamen University, Xiamen, China; ^2^School of Physical Education, Nanchang Normal University, Nanchang, Jiangxi, China; ^3^Shanghai Elite Sport Training Administrative Center, Shanghai, China

**Keywords:** APIM, partnership, expressive ties, competitive performance, athlete engagement

## Abstract

**Objectives:**

This study explores the significant impact of expressive ties (EI) between DanceSport couples on their competitive performance (CP). Utilizing a dyadic approach, we examined the performance achievement processes of DanceSport couples in relation to their EI.

**Methods:**

Participants comprised 67 dyads of Chinese elite dancers aged between 16 and 30 years. The dyadic analysis was carried out using a structural equation model based on the actor-partner interdependence mediation model.

**Results:**

With regard to actor effects, both male (β  =  0.292, *p* =  0.012) and female (β  =  0.443, *p* <  0.001) dancers’ perceived quality of EI had a positive correlation with CP. The males’ athlete engagement (AE) partially mediated the impact of EI on CP [indirect effect  =  0.144, SE  =  0.072, 95% confidence intervals (CI)  =  0.020, 0.283]. Regarding partner effects, females’ perceived EI quality positively influenced the male’s CP (β  =  0.26, *p* =  0.023) and mediated this association through the male’s AE [indirect effect  =  0.086, SE  =  0.041, 95% confidence intervals (CI)  =  0.003, 0.149]. Similarly, the females’ AE mediated the effect of males’ perceived EI quality on the females’ CP [indirect effect  =  0.152, SE  =  0.074, 95% confidence intervals (CI) =0.002, 0.256].

**Conclusion:**

We not only validated the propositions of the self-determination theory but also provided valuable insights to further enrich it. Our findings underscore that self-determination theory must account for individual gender characteristics.

## Introduction

Dance Sport, which is another name for competitive dance, is a stunning illustration of love desire. It is an open presentation of feelings, influenced by themes of sensuality and love (Harman, 2019). It emphasizes the intimate ballet between male and female partners, celebrating the pleasurable tension and harmony between them (Ericksen, 2011). DanceSport, a sophisticated form of male–female partnered dancing (Ogilvie, 2017, pp. 3), places romance as its utmost essence (John, 1998, pp. 11; Marion, 2014). Hence, the predominant aesthetic in partnered dance, particularly in ballroom, is the twinned collaboration between men and women (Marion, 2006; Budnik-Przybylska et al., 2015). In summarize, the cooperation between partners is the most important feature of sports dance, and emotional skills training is very important (Dan, 2012; Sun and Wu, 2023). So, the analysis of various problems surrounding DanceSport must always consider the partnership between couples (Pilewska et al., 2013). Yet, the connection between expressive ties and competitive performance in DanceSport pairs remains undefined. As a result, this research utilizes the Actor-Partner Interdependence Model (APIM) to delve into the association of expressive ties with competitive performance in DanceSport pairs and to assess gender differences. This could provide a theoretical framework for formulating distinctive interaction strategies for men and women in the future, as well as further enrich the theory of competitive behavior.

### Expressive ties between DanceSport dyads and competitive performance

Expressive ties, which incorporate two key factors – instant intimacy and long-term affection (Ericksen, 2011), play a pivotal role in influencing competitive performance (Liu et al., 2023). Instant intimacy is defined as a fleeting yet intense state of desire developed amidst the competition, forged between dance partners. It could stem from romantic attraction or a shared dedication to the dance form (John, 1998). Studies on event-related potentials (ERPs) have established that the brain’s electrical components react when individuals process information regarding others with whom they share a high level of intimacy. Notably, the N2 component (Chen et al., 2013), which signifies familiarity, and P3 (Matsunaga et al., 2012), associated with emotions, display elevated amplitudes. Moreover, fostering a potent sense of passion (Baumeister and Bratslavsky, 1999) is considered essential for excellent performance. High-level competitors often channel the intensity of romance between the sexes in dance by expressing passion for their partners, particularly during their performance (Liu and Wang, 2022; Liu et al., 2023). It is also finds that those with more frequent dance habits or elite dancers score higher on the expressive ties (Izountouemoi and Esteves, 2023).

The influence of instant intimacy on DanceSport competitive performance gains corroboration from neuroscientific research as well. The mirror neuron mechanism comes into play here, with specific regions of the human cerebral cortex, such as the inferior parietal lobule, the anterior Bocca region before the ventral motor, and the posterior segment of the inferior frontal gyrus exhibiting a “mapping” functionality of mirror neurons (Fogassi, 2011; Ye et al., 2016). This mapping mechanism translates the subtle sensual nuances of couples’ movements, like their entwined bodies, captivating eye-contact, synchronized breathing, and burgeoning passion, into emotional catalysts augmenting their partnership. The implications and significance of these minutiae of movements requires deeper perspective. DanceSport participants use a plethora of subtle to blatant cues to signify romantic interest, such as touching intimate body parts like the lower hips and buttocks, maintaining full-body contact, stroking the partner’s hair or face, dancing cheek to cheek, or maintaining intense eye contact, along with other unique gestures. (Joanna Bosse, 2015, pp. 61). Meneau (2020) provides an evocative description of an intimate dance movement: “He approaches her from behind until his chest touches her back, then thrusts his hands to her lower thighs and caresses her upwards. After grabbing her waist, he pushes her away and pulls her back to him, provoking an impact of her back against his ribcage.” Building on these sensual movements, Peters (1992) highlights the confusion dancers might experience due to the sexual tension on the dance floor, questioning the nature of their off-stage relationship. Moreover, Heyes and Catmur (2022) point out that mirror neuron-associated brain regions are crucial for simulating physical movements. Thus, when dancers showcase their sensual movements, the mirror neurons in their partners react, intensifying the intimacy of their performance.

Long-term affection signifies an emotional bond developed over prolonged interactions in both personal and professional spheres between dance partners. This varies from instant intimacy due to its more diluted emotional intensity and gradual emotional manifestation (Liu et al., 2023). Such long-term affection necessitates that the dancers’ esteem, care for, and harmonize with their partners. This is akin to the “partner care dimension” of DanceSport couples’ partnership (Myung et al., 2010), the “closeness dimension” in the 5C’s theory of athlete-athlete partnership (Poczwardowski et al., 2019), and also parallel to the 3C’s theory of the coach-athlete relationship (Jowett and Meek, 2000; Jowett and Cockerill, 2003; Jowett and Ntoumanis, 2004; Jowett and Poczwardowski, 2007; Jowett and Palmer, 2010). Key to maintaining a consistent training schedule, it paves the way towards achieving superior performance.

This may have its origins in the way that people are naturally “sociable,” a trait that society bestows on all people. Particularly for elite dancers, who often undertake training sessions over a decade or even two decades with same partner, this becomes salient. These dancers frequently relocate from their homes to other places for training, often independently without the backing of any organizations. So, they are compelled to support each other, practice tolerance, and forge a harmonious relationship model shaped by the trials of shared fortunes or misfortunes. Often, over this journey, they establish close emotional ties, such as romantic relationships.

When dancers feel understood and valued, their mutual intimacy escalates (Fitzsimons and Kay, 2004). Therefore, it becomes imperative for partners to exude both passion and emotion in their performance. It was frequently noted that classically trained dance couples also shared their lives off-stage (John, 1998, pp. 117; Majoross et al., 2008; Brewińska and Poczwardowski, 2012). A study discovered that among international professional dancers, a majority of couples were married and rarely described their bond as working relationships (Majoross et al., 2008). Additionally, research examining the social and psychological characteristics of dyadic participants revealed superior performances if the members fostered mutual likability (Krivonos et al., 1976).

### Mediating variable: athlete engagement

Interpersonal relationships provide the environment for cognitive sharing and performance enhancement (Davis et al., 2018; Staff et al., 2020). According to the motivation-hygiene theory, while interpersonal relationships serve as a hygiene factor that bolsters performance, under certain circumstances, they can transform into a motivating factor. This suggests the presence of mediators that facilitate the transition of interpersonal relationships into competitive performance-one of which is Athlete Engagement (AE).

Conceptualized from a positive psychology perspective by Lonsdale et al. (2007a,b), AE encompasses a persistent, positive, cognitive-affective state. It is characterized by the presence of confidence (belief in one’s ability to achieve high-performance levels and desired goals), dedication (the willingness to invest time and effort to fulfill important goals), vigor (a feeling of physical and mental vitality), and enthusiasm (intense excitement and enjoyment). Lonsdale et al. (2007a,b), based on the self-determination theory, proposed a strong correlation between relatedness and AE, a notion endorsed by multiple studies (Hodge et al., 2009).

Further supporting this, Ye et al. (2016) constructed a mediating model of AE and Hope, examining the relations between coach-athlete relationship and competitive performance satisfaction (which was self-reported due to the inconsistent performance scale across different sports events). Their study indicated a significant mediating effect of AE (β = 0.04, *p* < 0.001).

Motivated by this study, we propose that AE mediates the association between competitive performance and DanceSport partnerships. This creates a psychological environment conducive to the transformation of interpersonal relationships into competitive performance. Two reasons substantiate this hypothesis. First, according to the Self-Determination Theory and associated studies mentioned earlier, the fulfillment experienced in a DanceSport partnership, such as the quality of expressive ties, influences training quality and consequently, AE and competitive performance.

Second, studies reveal that athlete engagement, entailing facets like dedication, vigor, and confidence, impacts competitive performance. As observed by Tremayne and Ballinger (2008) in an interview with an Australian championship-level couple, judges tend to favor DanceSport couples who exhibit harmony, vigor, and confidence during competitions (sub-dimensions of AE). Moreover, high-performing dancers generally demonstrate diligence, a firm belief in their success, are goal-confident, and show higher motivation levels (Jackson and Beauchamp, 2011; Ifrar et al., 2020). Jackson et al. (2010) emphasized that sports partnerships are highly goal-oriented in terms of training and competition outcomes, and successful purposeful interaction hinges on joint and sustained dedication to practice, planning, and organization. Thus, desirable partner traits in these contexts include high conscientiousness (being reliable, disciplined, and dutiful). Furthermore, John (1998, pp. 33) asserted that successful DanceSport competitors are both gifted and dedicated athletes.

### Mutual influences between the DanceSport dyads and the applied of the APIM

Extant literature posits that interpersonal relationships are essentially dyadic in nature (Berscheid, 1999; Kivlighan, 2007; Reis, 2007; Ferris et al., 2009), with the dyadic structure forming the crux of these relationships (Reis and Collins, 2004). This structure encapsulates the fundamental attributes of interpersonal relationships. Firstly, it implies that the two parties involved are interdependent, considering this mutual dependence in planning their interactions and activities. Secondly, it denotes shared future expectations and distributed responsibilities among the dyads.

In the realm of DanceSport, exceptional performance hinges on the closeness of the partnerships, the mutual influence and dependence between partners, and their capacity to incite potent interpersonal feelings and cognition (Pistole, 2003; Ifrar et al., 2020). Thus, as partner dance forms, DanceSport dyads are thought to inspire each other’s emotions and behaviors (Middelberg, 2001). Despite previous studies considering the interaction between DanceSport partners, research on the interaction of such dyadic pairs has long been inadequate due to methodological limitations.

To elaborate, prior studies of dyadic relationships often attempted to analyze individual responses from each member, treating these as independent observations (e.g., analyzing 67 dyads as 134 individual cases) and predicting each individual outcome variable through their own predictor variables. This method overlooked the non-independent nature of dyadic data, thereby jeopardizing the veracity of the analysis (Kenny et al., 2006).

To address this, Kenny and his colleagues devised the Actor-Partner Interdependence Model (APIM; Kenny and Cook, 1999; Cook and Kenny, 2005). This model, an innovative approach to resolving issues of interdependence in dyadic research, distinguishes between partner effects (the influence of a partner’s characteristics on another’s attributes) and actor effects (the influence of one’s own characteristics on their attributes) (Carr, 2012, pp. 98). Through employment of this approach, numerous studies have begun focusing on interactions between dyadic couples.

However, a comprehensive review study revealed a significant dearth of research considering performance as an interpersonal outcome variable. It suggested that sports and workplace domains could redress this gap by fostering performance-enhancing psychological environments within the dyadic relationship sphere (Staff et al., 2017). In light of this, the present study attempts to validate an actor-partner interdependence mediation model, basing its construction on relevant literature.

### The current study: development of theoretical framework and hypotheses

DanceSport depictions lean extensively on heteronormative gender performances, featuring distinct romantic interactions between individuals of opposite sexes (Meneau, 2020). DanceSport couples fuse physical agility with artistic expression, giving prominence to emotional expression and mood creation. In fact, emotional expression between partners can often compensate for technical deficiencies (John, 1998, pp. 135). As a pair, dancers must collaborate and express their intentions through the directional flow of energy and pressure at different body points. Their elite performance hinges on their ability to cultivate and stimulate profound interpersonal feelings and cognition (Pistole, 2003). For dancers, the challenge lies within the synchronization of their movements and the atmosphere it generates (Nadel and Strauss, 2003). Therefore, it is proposed that the actor-partner effects of expressive ties significantly impact both male and female competitive performance.

This expression encompasses aspects such as “harmony/rapport,” “appreciation,” “care,” and “passion” between couples, deemed pivotal factors in DanceSport training. John (1998, pp. 27) sates, “harmony/rapport between the dancers is essential, be it driven by romantic attraction or a shared passion for the dance.” Mutual “appreciation” between dance partners refers to the shared adeptness in dance skills, serving as a crucial predictor of their competitive performance.

Enduring interaction over time necessitates DanceSport dyads to exhibit “care” towards each other, which aids in achieving win-win competitive outcomes. Furthermore, “passion” between dancers signifies an optimal competitive condition that appeals to the judges, thus securing a favorable score and culminating in superior competitive performance. Even though competition scoring criteria require judges to rate dancers based on body control, posture, shape, footwork, timing, rhythm, and the complexity of the routine (Pittman et al., 2005), these aspects must be evaluated within a limited time frame in an environment where up to 50 couples compete in the initial rounds with judges eliminating 50% of the couples in 2 min.

Achieving excellent competitive performance extends beyond technical accuracy. The crux lies in presenting a harmonious, elegant, and captivating overall impression (Tremayne and Ballinger, 2008). Fostiak (1996) reinforces this by encouraging dancers to “feel your partner,” promoting harmony with their partners. Hence, we propose:

*H1:* male’s perceived expressive ties quality will influence his own competitive performance.

*H2:* female’s perceived expressive ties quality will influence her own competitive performance.

*H3:* male’s perceived expressive ties quality will influence female’s competitive performance.

*H4:* female’s perceived expressive ties quality will influence on male’s competitive performance.

**Figure 1:**
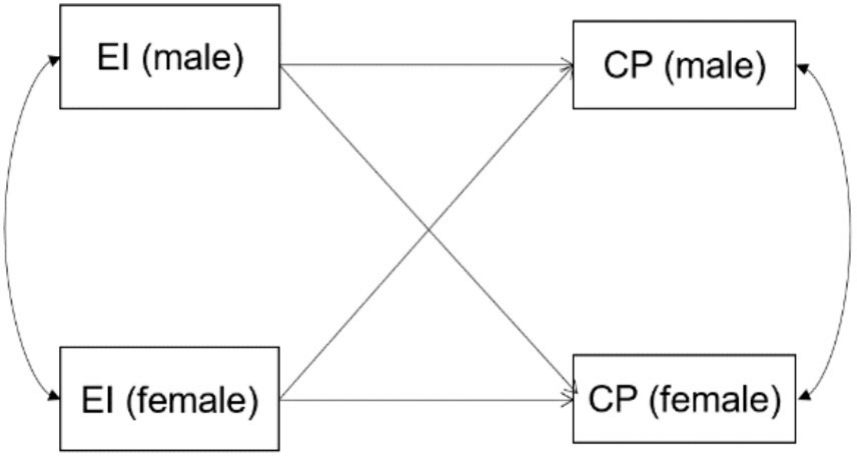
The actor-partner effect hypothetical model of athlete dyads’ expressive ties on their own and partners’ competitive performance.

The hypothesis above forms the model shown in Figure 1.

Under the mechanism of mirror neurons, partners exhibit their innate physiological responses during cooperation and interaction, specifically, sexual attraction and flirtation. This sparks a strong short-term passion between dance partners which fuels both their own and their partner’s enthusiasm for dance. This non-cognitive element significantly impacts competitive performance. Furthermore, high-quality expressive ties encourage more frequent cooperation between partners, enhancing individual competitive performance and satisfaction with competitive performance. Thus, both males and females can augment their athletic enthusiasm and individual competitive performance through the intense passion displayed on the competition court and their expressions of appreciation, care, and so on. As a result, we put forward the following proposition:

*H5:* male’ athlete engagement partially mediated the effect of his own perceived expressive ties quality on his own competitive performance.

*H6:* female’ athlete engagement partially mediated the effect of her own perceived expressive ties quality on her own competitive performance.

*H7:* male’ athlete engagement partially mediated the effect of female’s perceived expressive ties quality on male’s competitive performance.

*H8:* female’ athlete engagement partially mediated the effect of male’s perceived expressive ties quality on female’s competitive performance.

**Figure 2:**
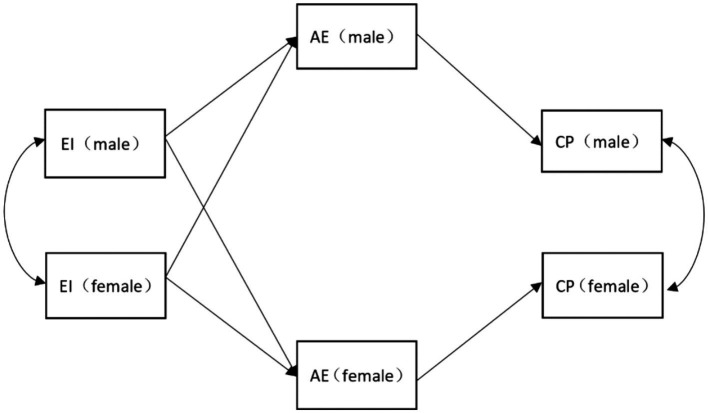
The mediated hypothesis model of the effect of expressive ties between couples on competitive performance.

The hypothesis above forms the model shown in Figure 2.

## Methods

### Procedure

This study adopted a cross-sectional design and upon receiving institutional ethical approval, participants were canvased via email to partake in the study. Participants received standardized information elucidating the purpose of the study and pertinent ethical considerations including confidentiality, anonymity, the right to withdraw, and data protection.

### Participants

Our sample comprised of 134 participants (67 dyads) who took part in the 2019 Chinese DanceSport Championship (Beijing Station), the apex event in Chinese DanceSport hosted at Ditan Gymnasium. We engaged three experienced international-level DanceSport judges to choose the participants for this study based on the competitors’ performance abilities. Thus, a pool of 242 competitors was initially selected. The criteria for inclusion included: (a) having a regular partner for at least three years; (b) show of exemplary performance in the past. To minimize distortion due to athletic ability, a second round of screening was carried out, resulting in the selection of 134 participants (67 dyads) with comparable competitive levels for the study.

By proactively communicating with participants as well as their coaches or friends, we secured their trust and backing. After the competition, paper questionnaires were distributed right away and electronic versions were given to those who preferred to fill them out later due to the noisy venue and physical exhaustion from competition. Two researchers assisted with data collection to ensure the independence of each person’s responses. The participants’ average age was 19.85 for males (SD = 4.02) and 18.94 for females (SD = 3.07). Among male dance partners, 26 believed they had formed a strong friendship with their female partners and more considered them as close relatives (5) and lovers (17) compared to the females (22, 4, 15). However, 26 female dance partners felt they had strictly cooperative partnerships with their male counterparts, a sentiment shared by 19 male dance partners.

## Materials

### Expressive ties quality

The Expressive Ties in the Partnership Scale-DanceSport Couples (PS-DSC) was adopted for the current research (Liu and Wang, 2022; Liu et al., 2023). The questionnaire consists of 4 items, namely “In dance training or competition, I and my partner were full of passion,” “I get along with my partner,” “I and my partner appreciate each other,” “I and my partner care about each other” and Cronbach α was 0.924. Confirmatory factor analysis (CFA) was conducted to examine the validity of the measurement.

### Athlete engagement

Athlete Engagement Questionnaire (AEQ) (Lonsdale et al., 2007a,b) was adopted for our study. The good adaptability of the questionnaire among Chinese athletes has been verified (Wang et al., 2014; Ye, 2014; Ye et al., 2016). The scale consists of sixteen items, and Cronbach α was 0.943, and its’ four dimensions Cronbach α was 0.867, 0.884, 0.910, 0.841, respectively.

### Competitive performance

Performance includes satisfaction of it and competition field performance, so the Competitive Performance Questionnaire was with 4 items in total. (1) Athlete Satisfaction Questionnaire (ASQ) (Riemer and Chelladurai, 1998) was adopted. (2) the ranking, which was ordinal variable, in the competition was the performance outcomes, so it was assigned to a 5-level Likert scale according to 5 international-level DanceSport judges, the results obtained after data processing of five levels of ordinal variables with continuous variables were not biased. The scale consists of 4 items, and Cronbach α was 0.702. Confirmatory factor analysis (CFA) was conducted to examine the validity of the measurement. The fit indices were: *χ^2^/df =* 0.042*, NFI* = 0.999, AG*FI* = 0.998, *PNFI* = 0.333, *RMSEA* = 0.000, *SRMR* = 0.005.

### Analysis strategy

We utilized SPSS 22.0 and Mplus 8.3 for data processing and analysis. To begin with, we performed a descriptive statistical analysis to calculate the mean, standard deviation, and correlation of all variables in order to gain insights into the characteristics of variables and their interrelationships. Subsequently, following the process outlined by Davis et al. (2013), we implemented the actor and partner interdependence model to scrutinize the reciprocal impact of one’s expressive ties with their partner on their own competitive performance, as well as that of their partner’s (Kenny et al., 2006). This statistical method, suitable for dyadic data analysis, has been extensively used in intimate-relationship studies (Campbell and Kashy, 2002). More specifically, the actor effect relates to the influence of an individual’s characteristics on their own outcome variables, while the partner effect pertains to the impact of an individual’s variables on their partner’s outcome variables. Additionally, before analyzing the actor-partner effects, we will test the hypothesized model using the following indices: χ^2^, df, CFI, TLI, RMSEA, SRMR. If the conditions of CFI > 0.9, TLI > 0.9, RMSEA<0.08, and SRMR<0.08 (Hu and Bentler, 1999) are satisfied, the model would be considered acceptable.

## Results

Table 1 illustrates that for both genders, competitive performance is significantly linked not only with one’s own perception of the quality of expressive ties but also with one’s partner’s perceived quality of expressive ties. More strikingly, the correlation coefficients for the quality of expressive ties in females and the competitive performance of males (r = 0.376, *p* < 0.01) exceeded those for the quality of expressive ties in males and the competitive performance of females (r = 0.393, *p* < 0.01). The scores of athlete engagement for both females and males showed a positive correlation with their respective competitive performances.

**Table 1:** Mean, standard deviation, and correlation matrix of each research variable.

	M	SD	1	2	3	4	5	6
1. Expressive ties (M)	15.87	3.40	1					
2. Athlete Engagement (M)	70.40	9.69	0.454^***^	1				
3. Competitive performance (M)	12.19	3.59	0.393^**^	0.440^***^	1			
4. Expressive ties (F)	14.67	4.06	0.378^**^	0.266^*^	0.376^**^	1		
5. Athlete Engagement (F)	66.85	9.94	0.458^**^	0.281^*^	0.303^*^	0.448^***^	1	
6. Competitive performance (F)	12.00	2.88	0.269^*^	0.230	0.517^***^	0.482^***^	0.473^***^	1

*, *p* < 0.05; **, *p* < 0.01; ***, *p* < 0.001. *N* = 134 couples; F, female; M, male.

Moreover, we employed the Actor-Partner Interdependence Model (APIM) to analyze the mutual influence between the quality of expressive ties and competitive performance, moderated by the level of athlete engagement. Initially, to decrease covariance for a clearer understanding of the intercept before establishing the APIM, each independent variable needed to undergo a central transformation. This transformation was achieved by subtracting the score of expressive ties and athlete engagement from the overall sample mean of that variable (He et al., 2018). In order to verify the actor-partner effect of paired competitors’ own expressive ties on their own or their partner’s competitive performance, this study utilized the expressive ties between the couples as independent variables in Mplus, and their competitive performance as the dependent variables for model testing.

**Figure 3:**
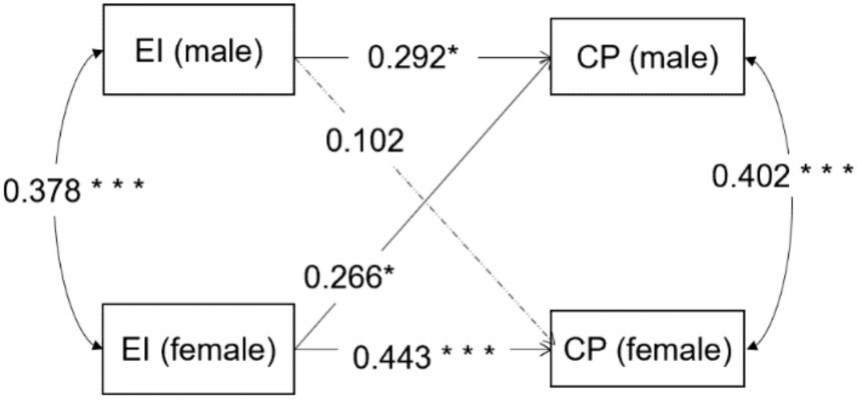
The actor-partner effect model of athlete dyads’ expressive ties on competitive performance.

In Figure 3, the actor-partner effect model of athlete dyads’ expressive ties on competitive performance was a saturated model (*χ^2^ = 0*, *df* = 0 and the parameters to be estimated in the model were exactly equal to the elements in the covariance matrix) which means that the model was just identified and has a perfect fit. In this case, the model fit index was no longer reported and only the path coefficients need to be focused on (Li et al., 2019).

As shown in Table 2, regarding the actor effects, male’s perceived expressive ties quality (*β =* 0.292, *p* = 0.012) and the female’s (*β =* 0.443, *p* < 0.001) will influence their own competitive performance. Regarding the partner effects, female’s perceived expressive ties quality exerts influence on male’s competitive performance (*β =* 0.266, *p* = 0.023). However, male’s perceived expressive ties quality cannot influence female’s competitive performance (*β =* 0.102, *p* = 0.374). So, hypothesis H1, H2, H4 were supported by our study, but H3 was unsupported.

**Table 2:** Standardization path coefficient and hypothesis testing results of expressive ties’ impact on competitive performance.

Effect	Path	*β*	*t*	*p*	Test results
Actor effect	ET (M) → CP (M)	0.292	2.498	0.012	H1: supported
Actor effect	ET (F) → CP(F)	0.443	3.854	<0.000	H2: supported
Partner effect	ET (M) → CP (F)	0.102	0.889	0.374	H3: unsupported
Partner effect	ET (F) → CP (M)	0.266	2.276	0.023	H4: supported

ET, expressive ties; CP, competitive performance; F, female; M, male.

**Figure 4:**
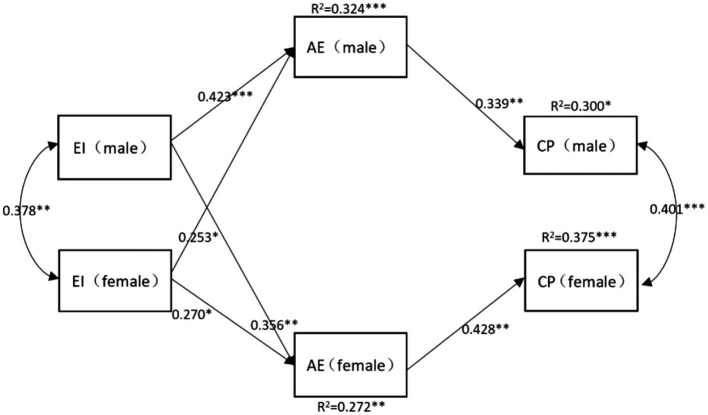
The mediating model highlighting the influence of expressive ties on competitive performance.

Then, we add the athlete engagement as the mediator in the model, the mediating effect was analyzed using the bootstrap method, and 5,000 Bootstrap runs were performed, and the mediating effect was significant if the 95% CI did not contain 0. Figure 4 showed the mediating model, Table 3 showed the fitting index of the model. The model was perfect.

**Table 3:** Intermediate model fitting of dance partnership expressive ties on competitive performance.

	*χ^2^*	*df*	CFI	TLI	RMSEA	SRMR
AE	115.934	14	1.000	1.020	0.000	0.017

As shown in Figure 4, male’s perceived expressive ties quality will influence his own athlete engagement (*β* = 0.423, *p* < 0.001) and female’s athlete engagement (*β* = 0.356, *p* = 0.006). Female’s perceived expressive ties quality has marginal significant effect on her own athlete engagement (*β* = 0.270, *p* = 0.076), and will influence male’s athlete engagement (*β* = 0.253, *p* = 0.011). Male’s athlete engagement level will influence his own competitive performance (*β* = 0.339, *p* = 0.005), female’s athlete engagement level will influence her own competitive performance (*β* = 0.428, *p* = 0.002).

As shown in Table 4, regarding the actor effects, male’ athlete engagement partially mediated the effect of his own perceived expressive ties quality on his own competitive performance [indirect effect = 0.144, *SE* = 0.072,95% confidence intervals (*CI*) = 0.020, 0.283]. However, female’ athlete engagement cannot mediate the effect of her own perceived expressive ties quality on her own competitive performance [indirect effect = 0.152, *SE* = 0.069,95% confidence intervals (*CI*) = −0.004,0.168]. Regarding the partner effects, male’ athlete engagement partially mediated the effect of female’s perceived expressive ties quality on male’s competitive performance [indirect effect = 0.086, *SE* = 0.041, 95% confidence intervals (*CI*) = 0.003, 0.149]. in addition, female’ athlete engagement partially mediated the effect of male’s perceived expressive ties quality on female’s competitive performance [indirect effect = 0.152, *SE* = 0.074,95% confidence intervals (*CI*) =0.002, 0.256]. the H5, H7, H8 were supported by our study, but H6 was unsupported.

**Table 4:** Significance test results of bootstrap mediation effect of expressive ties on competitive performance.

Path	Effect value	Bootstrap SE	95% Bootstrap Cl	Test results
ET(M) → AE(M) → CP(M)	0.144	0.072	[0.020, 0.283]	H5: supported
ET(F) → AE(F) → CP(F)	0.115	0.069	[−0.004,0.168]	H6: unsupported
ET(M) → AE(F) → CP(F)	0.152	0.074	[0.002, 0.256]	H7: supported
ET(F) → AE(M) → CP(M)	0.086	0.041	[0.003, 0.149]	H8: supported

ET, expressive ties; AE, athlete engagement; CP, competitive performance; F, female; M, male.

## Discussion

### Actor effect analysis

Our results indicates that male’s **(H1)** and female’s **(H2)** perceived expressive ties quality affects their own competitive performance, and male’s athlete engagement partially mediates the effect of his own perceived expressive ties quality on his own competitive performance **(H5)**. These findings demonstrates that expressive ties between DanceSport partners and athlete engagement are critical factors influencing individuals’ competitive performance. The findings of this study align coherently with the principles of Self-Determination Theory (SDT), as introduced in the context of the associated applied research. Partners engaging in nascent relational dynamics have been shown to exhibit increased levels of oxytocin (Moll et al., 2010), a biochemical change that can extend into the athletic milieu, potentially yielding a positive influence on performance (Campbell et al., 2016). Furthermore, Campbell et al. (2016) assert that while expressive relational ties can have beneficial spillover effects that enhance athletic outcomes, concomitant negative ramifications are plausible as per Reis and Aron (2008). Specifically, early-stage romantic involvements may be fraught with elements such as jealousy or conflict, precipitating fluctuations in mood, and episodes of anxiety and depression among partners. The spectrum of these emotional disturbances, irrespective of their perceived severity, holds the capacity to directly compromise an athlete’s performance. However, for the present study, the dancers tend to be more in a cooperative relationship, with the referee scoring for dance dyads rather than one of them. Perhaps it is precisely because of this that dancers will feel happy rather than jealous and make appointments due to the progress of their partners, thus generating an actor effect, making highly expressive ties positively affect the dancer’ competitive performance.

However, our results show that female’ athlete engagement cannot mediate the effect of her own perceived expressive ties quality on her own competitive performance **(H6)**, which is inconsistent with the general interpersonal interaction (Kandel, 1978). The reasons may be as following:

1. There is a threshold for female’s athlete engagement to be activated to frame a mediating path between expressive ties and competitive performance, but the level of expressive ties quality that exists in and out of the arena from females to males is not sufficient to activate female’s athlete engagement. On one hand, physiological load on females are more intensity than on males. For example, A study simulating competition situation found that, based on the characteristics of special holding technology in the modern dance, the HR_sim_/HR_max_ and VO_2hold_/VO_2max_ of males was significantly lower than that of females (*p* < 0.01), and pointed out that the oxygen consumption of females was 78% of that of male (*p* < 0.05), and the heart rate would increase by 14% (*p* < 0.05) when maintaining holding posture (Vaczi et al., 2016). According to sports physiology, overcoming the physiological load means that females need to have more positive psychological characteristics, and athlete engagement is the psychological characteristic required in sports, especially among elite athletes. We believe that, to a certain extent, females need to complete the competition through more confident, enthusiastic, and other positive high-level elements of athlete engagement. On the other hand, Females are more introverted and tend to avoid intimacy (Guo, 2016). Influenced by the traditional Chinese cultural, based on the gender theory, females will not express their hot thoughts too much. For example, in our study, while some males assess their female partners as lovers, females only perceived the existence of a cooperative relationship with them.
2. Females’ athlete engagement (e.g., passion and confidence) may not be strong predictors of their own competitive performance. Although females’ technical movements in the arena are fuller of desire and are more likely to infect the audience and judges according to common sense, however, an empirical study proves that the passion of males will influence judges and audiences, while females’ passion cannot (Yang, 2016). This is in line with the gender theory, gender segregation and stratification are worldwide phenomena, although more women have entered traditionally male occupations, so gender stratification will decrease. However, the road to the top of the professional level is not always smooth. Although not all females are excluded, they find it difficult to pass the middle tier in the professional field. The common and in-depth phenomenon that females blocked on the way forward is called glass ceiling: females can see her goals, but they will hit an obstacle that is invisible and cannot be passed through. In a survey of deputy directors of female cooperation, 71% of people said that their organizations have glass ceilings for females. However, 73% of male directors in the unified organization believed that this did not exist. This may be, as Williams and Best believed in a 1982 study in 30 countries, that gender stereotypes had been formed before the age of five, which developed rapidly in early school years and completed fully in adolescence, so many people were not aware of their existence (Crawford and Unger, 2009, pp. 677–680).

### Partner effect analysis

Our results show that female’s perceived expressive ties quality affects male’s competitive performance **(H4)**; male’ athlete engagement partially mediates the effect of female’s perceived expressive ties quality on male’s competitive performance **(H8)**. This finding demonstrates that male’s athlete engagement is an important mediator. And this finding is in line with the ideas mentioned by SDT which states that athlete engagement put great influence in competitive performance, and also be verified by scholars that males may have a sense of unity and dominance with females after being infected by their enthusiasm, vitality and other athlete engagement characteristics (Lin, 2008), which means that the males were more passionate, and as Majoross et al. (2008) stated that the males’ passion will have a critical impact on the judges and the audiences. To be more specific, according to Strathern (1996), dancers’ physical expression needs to consider gender values, gender settings of DanceSport, etc. On one hand, the dualistic framework of gender roles (a dualistic concept called “*yin*” and “*yang*” in Chinese philosophy) cast the gender characteristics of strong males and weak females (Lin, 2008). On the other hand, because male dominance and female obedience occupies the core of western romantic impression and modern dating stereotype (Crawford and Unger, 2009, pp. 174), therefore, the technical characteristics of DanceSport require males to guide and females to follow. The dominant position of male requires them to conduct image training on the venue and dance before the competition. They imagine themselves to be strong and tough and expect themselves and their partners to regard each other as their lovers, thus creating a sense of unity. Even after finishing the dance competition, males feel that they are a proud lion who won the competition (Lin, 2008).

In addition, female’s athlete engagement partially mediates the association between male’s perceived expressive ties quality and female’s competitive performance **(H7)**. It may be that the males’ perceived expressive ties quality (e.g., care, appreciation, and passion) stimulates the females’ athlete engagement and reaches a certain threshold, which would help predict the females’ competitive performance. Specifically, after sharing the experience of weal and woe with females, males form a high perceived expressive ties quality. As shown by the matching characteristics of dance partners in our study, the average value of males in the expressive ties is 15.87, which is higher than the average value of females (14.67). When females have not recognized the relationship between them as a couple, males believe that they have reached a romantic relationship with females. This means that males have stronger emotions such as long-term care, appreciation, as well as stronger passion on the competition context. This can help males strengthen their self-concept, generate a state of confidence, vitality, and enthusiasm, and be more confident to succeed with their partners. Confidence is an important connotation of engagement, and is one of the most important abilities for high-level athletes to achieve successful performance (Platonov, 2014).

In addition, the contradiction between the results of H8 and H7 is that high expressive ties in males may not promote females’ competitive performance, for males tend to perform worse in expressive ties, this may be detrimental to the emotional satisfaction of female Dance partners (Niedźwieńska and Zielińska, 2020), and according to self-determination theory, this may also result in high expressive ties in males not promoting female dance partners’ competitive performance and athlete engagement. Further empirical research is needed to verify whether this result can be extended to DanceSport couples.

Our study also finds that male’s perceived expressive ties quality cannot affect female’s competitive performance (**H3**), which does not conform to Donohue et al. (2007)’s viewpoint, that is supportive relationships help bolster performance. The reason may be as following: males are more confident and open as mentioned above, which make them overestimate their perceived expressive ties quality, thus making it difficult to predict the female’s competitive performance. Especially attitude is heightened in Latin dances, this is no place for the timid, especially for male dancers, whose manner must be confident to the point of domination (John, 1998, pp. 40). In addition, based on gender stereotypes, males have a deep-rooted sense of dominance, and their level of self-confidence and work engagement is higher than that of females (Ackerman et al., 2011). A survey of Canadian public officials proves this view (Rabinowitz and Hall, 1977). So, it is not surprising, in our study, that males believe that females are their girlfriends on the premise that females do not recognize the intimate relationship between them. This more “aggressive” tendency may lead to a miscalculation of the current training or competition situation.

## Conclusion

The findings of this study not only affirm the assertions of the Self-Determination Theory (SDT), but significantly contribute to its enrichment and enhancement. In particular, this calls for the SDT to consider individual gender characteristics. Specifically, (1) fulfillment of related sense or athlete engagement impacts performance output. Our research showed that males’ and females’ perceived expressive ties quality cast an actor effect on their own competitive performance, and male athlete engagement partially intervened in the correlation between his perceived expressive ties quality and his performance. Moreover, females’ perceived expressive ties quality project a partner effect on males’ performance, with male athlete engagement partially intervening in this correlation, and female athlete engagement partially intervening in the effect of male’s perceived expressive ties quality on female’s performance. (2) Over satisfaction of self-related sense or athlete engagement does not necessarily enhance performance output because males’ perceived expressive ties quality, influenced by gender role traits imposed by Chinese cultural traditions and competitive sports culture, might intensify athlete engagement to a point resulting in misjudgment of current training or competition situations, and possibly fail to objectively promote female partners’ performance, hence, our study shows male’s perceived expressive ties quality has no significant impact on female’s performance. Furthermore, female athlete engagement did not mediate the effect of her own perceived expressive ties quality on her performance, potentially because a threshold exists for female athlete engagement to facilitate a role in their own competitive performance, or due to biases in the DanceSport scoring system. Hence, we propose that both genders should foster their own as well as their partner’s athlete engagement, which mediates the influence of expressive ties on competitive performance. Males should work diligently towards enhancing female partners’ athlete engagement (e.g., elevating confidence and passion), while females should also take initiatives to develop their engagement. Simultaneously, males need to establish expressive contact with females more objectively.

## Strengths and limitations

This study is framed by its strengths and limitations. The two noteworthy strengths are: (1) By embracing the essence of DanceSport, a dance form that revolves around love, gender (Ericksen, 2011, p. xii), and a romantic fantasia (Harman, 2019), we assume that the dynamics between DanceSport pairs significantly influences competitive outcomes. Our study explores this through the novel actor–partner interdependence mediation model to comprehend the mechanics of mixed-gender cooperation on performance, emphasizing male and female dancers’ mutual influences. Earlier investigations have rarely scrutinized these dynamics, and even when considering paired sporting activities, the dominant focus is either related to athletic performance or sustains a descriptive account of observations on how partnering impacts results. (2) The conclusions of this study add robustness to the scientific aspects of the Self-Determination Theory (SDT), and contribute to its development by bringing individual gender attributes into focus, a significant aspect for the comprehensive evolution of SDT.

Nonetheless, the embryonic nature of our study brings forth two limitations: (1) Our sample is exclusively drawn from Chinese dance pairs, which limits the global generalizability of our findings. It would be beneficial for further research to engage broader samples from different countries. (2) In addition, the sample size warrants expansion. Although similar studies have been conducted with less than 67 pairs (e.g., Jackson et al., 2010 with 58 pairs, Habeeb et al., 2017 with 51 pairs) statistical errors are potential risks. For future studies, enlarging the sample size is an important consideration. Although expanding the sample size beyond 240 is challenging due to the constraints of competitions and performance information, along with the need to control the impact of variables such as referees, lighting, and venue, it is important for future research to consider.

## Future research directions

Recognizing the cultural fabric woven into interpersonal interactions, future research needs to investigate the cultural context of dance partnerships (as unique relationships), delve deeper into their mutual influences, and perpetually enhance the theoretical frame of dance partnerships and competitive performance. In particular, Hsu (1953) suggests an exploration of interpersonal relationships within certain contexts. Furthermore, aligning with the epistemological strategy of cultural psychology, which echoes with the concept of “one mind, many meanings; disunified universalism” (Shweder et al., 1998, pp. 871; Hwang, 2018), and incorporating available studies on DanceSport partnerships and dance partner interaction practices, it becomes evident that DanceSport partnerships exhibit cultural identities (Liu et al., 2023). Thus, it becomes essential to thoroughly examine the backdrop of how dance partnerships impact competitive performance.

## Data availability statement

The original contributions presented in the study are included in the article/supplementary material, further inquiries can be directed to the corresponding author.

## Ethics statement

The studies involving humans were approved by the Institutional Review Board of Nanchang University. The studies were conducted in accordance with the local legislation and institutional requirements. Written informed consent for participation was not required from the participants or the participants' legal guardians/next of kin in accordance with the national legislation and institutional requirements.

## Author contributions

XL: Conceptualization, Data curation, Formal analysis, Funding acquisition, Investigation, Methodology, Project administration, Resources, Software, Supervision, Validation, Visualization, Writing – original draft, Writing – review & editing. YL: Writing – review & editing. DP: Writing – review & editing. XW: Supervision, Writing – review & editing.
